# *In silico* structural homology modeling and functional characterization of *Mycoplasma gallisepticum* variable lipoprotein hemagglutin proteins

**DOI:** 10.3389/fvets.2022.943831

**Published:** 2022-08-04

**Authors:** Susithra Priyadarshni Mugunthan, Mani Chandra Harish

**Affiliations:** Department of Biotechnology, Thiruvalluvar University, Vellore, India

**Keywords:** variable lipoprotein hemagglutin, immune evasion, bioinformatics, avian mycoplasmosis, *M. gallisepticum*

## Abstract

*Mycoplasma gallisepticum* variable lipoprotein hemagglutin (vlhA) proteins are crucial for immune evasion from the host cells, permitting the persistence and survival of the pathogen. However, the exact molecular mechanism behind the immune evasion function is still not clear. *In silico* physiochemical analysis, domain analysis, subcellular localization, and homology modeling studies have been carried out to predict the structural and functional properties of these proteins. The outcomes of this study provide significant preliminary data for understanding the immune evasion by vlhA proteins. In this study, we have reported the primary, secondary, and tertiary structural characteristics and subcellular localization, presence of the transmembrane helix and signal peptide, and functional characteristics of vlhA proteins from *M. gallisepticum* strain R low. The results show variation between the structural and functional components of the proteins, signifying the role and diverse molecular mechanisms in functioning of vlhA proteins in host immune evasion. Moreover the 3D structure predicted in this study will pave a way for understanding vlhA protein function and its interaction with other molecules to undergo immune evasion. This study forms the basis for future experimental studies improving our understanding in the molecular mechanisms used by vlhA proteins.

## Introduction

The bacteria of class Mollicutes are described as simplest self–replicating life forms due to their small cell size and complete lack of cell wall, limited metabolic pathway and reduced genome size (1). The *Mycoplasmataceae* family in Mollicutes includes majority of disease causing pathogens in medical and veterinary fields. A great number of *Mycoplasma* species are pathogenic to humans and animals which cause chronic infections consequential in infectious diseases. To adapt and survive the challenging and complex host environment, the mycoplasmas use combinational genetic machinery for phase and size variation of major surface components. Due to the lack of cell wall, the outer surface of the mycoplasma membrane plays a crucial role in the infection process, transport of nutrients, interaction with host cells, and host immune defense. Thus, gaining knowledge in the process of how and when the antigenic variation occurs can offer important insights to the tactics used by mycoplasmas to cause infection in host cells.

*Mycoplasma gallisepticum* is one of the most important avian pathogens which causes chronic respiratory disease (CRD) in chickens with the symptoms of cough, nasal discharge, low appetite, reduced hatchability and chick viability, loss of weight, and decreased egg production (1, 2). The responsible pathogenic events are due to genes that encode cytoadhesion and surface components with antigenic variation which involves the immune evasion of the host (3). *M. gallisepticum* infection results in infectious sinusitis in turkeys (swollen infraorbital sinuses) and conjunctivitis in finches.

**Figure 1:**
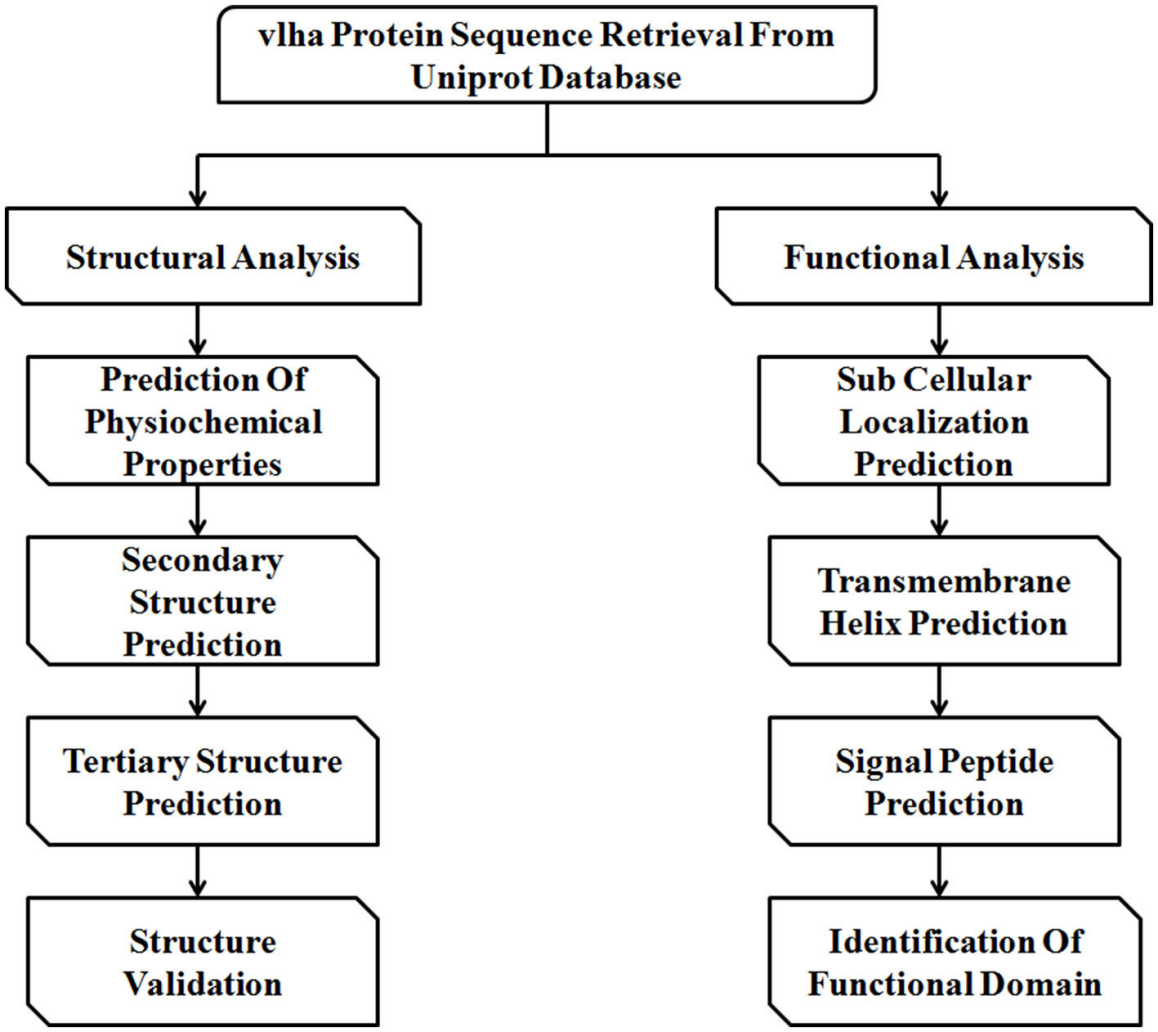
Schematic representation of the workflow followed in this study.

The immune evasion of *M. gallisepticum* is regulated by the vlhA gene family. This family consists of 43 vlhA genes located in five loci (Table 1). The major function of this gene family is to engender antigenic diversity which assists in immune evasion during infection. The vlhA gene family shows phase variation during acute phase and immune evasion during the chronic phase of infection (4, 5). The phase variation may occur impulsively or by an immune attack and is crucial for survival of *M. gallisepticum* in host cells (6–8). Various mechanisms for phase variation like gene conversion, site specific recombination, DNA slippage, and reciprocal recombination were utilized by different species of Mycoplasma (9). The vlhA gene products are speculated to be engaged in the attachment of host apolipoprotein A1 (10, 11) and red blood cells (12). The phase variation of *M. gallisepticum* is exclusive and has not been studied yet. Among the other vlhA genes, vlhA 3.03, 2.02 and 4.01 genes are primarily expressed in the initial phase of infection, whereas vlhA 1.07 and 5.13 are expressed in the later stages of infection. The prototype followed by *M. gallisepticum* to express the dominant vlhA gene during the course of infection is stochastic and the mechanism is unknown and yet to be explored (4). This study employed computational tools to understand the evolutionary relationship of the vlhA proteins; structural studies which include its primary sequence analysis, and secondary and tertiary structural analysis, functional studies like the cellular localization, presence of the transmembrane helix and signal peptide in vlhA proteins, and finally identification of functional domain were performed. To date, no *in silico* structural and functional studies have been reported for *M. gallisepticum* vlhA proteins. The diagrammatic representation of the work flow is presented in Figure 1. The list of bioinformatics tools and servers employed in this study is given in Table 2.

**Table 1:** List of vlhA genes based on their group analyzed in this study.

**vlhA 1**	**vlhA 2**	**vlhA 3**	**vlhA 4**	**vlhA 5**
vlhA.1.01 vlhA.1.02 vlhA.1.03 vlhA.1.04 vlhA.1.05 vlhA.1.06 vlhA.1.07 vlhA.1.08 vlhA.1.08b	vlhA.2.01 vlhA.2.02	vlhA.3.01 vlhA.3.02 vlhA.3.03 vlhA.3.04 vlhA.3.05 vlhA.3.06 vlhA.3.07 vlhA.3.08 vlhA.3.09	vlhA.4.01 vlhA.4.02 vlhA.4.03 vlhA.4.04 vlhA.4.05 vlhA.4.06 vlhA.4.07 vlhA.4.07.1 vlhA.4.07.2 vlhA.4.07.4 vlhA.4.07.6 vlhA.4.08 vlhA.4.09 vlhA.4.10 vlhA.4.11 vlhA.4.12	vlhA.5.01a vlhA.5.01b vlhA.5.01c vlhA.5.02 vlhA.5.03 vlhA.5.04 vlhA.5.05 vlhA.5.06 vlhA.5.07 vlhA.5.08 vlhA.5.09 vlhA.5.10a vlhA.5.10b vlhA.5.11 vlhA.5.12 vlhA.5.13

**Table 2:** List of bioinformatics tools and servers employed in the structural and functional analyses of vlhA proteins.

**S. no**	**Characterization/ analysis**	**Name of the server/tool**	**URL**
1.	Phylogenetic analysis	Phylogeny.fr	http://www.phylogeny.fr/simple_phylogeny.cgi
2.	Physiochemical properties	ExPASy-Protparam tool	https://web.expasy.org/protparam/
3.	Secondary structure	SOPMA	https://npsa-prabi.ibcp.fr/cgi-bin/npsa_automat.pl?page=/NPSA/npsa_sopma.html
		GOR IV	https://npsa-prabi.ibcp.fr/cgi-bin/npsa_automat.pl?page=npsa_gor4.html
4.	Tertiary structure	Raptor X	http://raptorx.uchicago.edu/
		I Tasser	https://zhanglab.dcmb.med.umich.edu/I-TASSER/
5.	Structure validation	PROCHECK	http://www.ebi.ac.uk/thornton-srv/databases/pdbsum/Generate.html
		QMEAN	https://swissmodel.expasy.org/qmean/
6.	Sub cellular Localization	PSLPRED	http://crdd.osdd.net/raghava/pslpred/
		PSORTB	https://www.psort.org/psortb/
		CELLO2GO	http://cello.life.nctu.edu.tw/cello2go/
7.	Transmembrane Helix	SOSUI	https://harrier.nagahama-i-bio.ac.jp/sosui/mobile/
		HMMTOP	http://www.enzim.hu/hmmtop/
		TMHMM	http://www.cbs.dtu.dk/services/TMHMM/
8.	Signal peptide	Signal p	http://www.cbs.dtu.dk/services/SignalP/
		Target p	http://www.cbs.dtu.dk/services/TargetP/
9.	Functional Domain	CDD- BLAST	https://blast.ncbi.nlm.nih.gov/Blast.cgi
		HmmScan	https://www.ebi.ac.uk/Tools/hmmer/search/hmmscan
		Pfam	http://pfam.xfam.org/
		SCANPROSITE	https://prosite.expasy.org/scanprosite/
		SMART	http://smart.embl-heidelberg.de/

Understanding the structural and functional properties of vlhA proteins of *M. gallisepticum* will provide the first step/lead in the direction of understanding of underlying molecular mechanisms involved. In this study, we used *in silico* methods to determine the physical, structural, and functional characteristics of vlhA proteins.

## Materials and methods

### Sequence retrieval

The amino acid sequences of vlhA proteins from *Mycoplasma gallisepticum* strain R low used in this study were retrieved from UniProt in the FASTA format. The protein names and their unique UniProt IDs are shown in Supplementary Table 1.

### Phylogenetic analysis

To understand the evolutionary relationships between the vlhA proteins, a phylogenetic tree was constructed using Phylogeny.fr, online software for phylogenetic analysis (13). The “One Click” option was used where the alignment was performed by MUSCLE, curation was performed by Gblocks, phylogeny was performed by PhyML, and Tree Rendering was performed by TreeDyn.

### Structural analysis

#### Physiochemical properties

The ExPASyProtparam tool was used to analyze the physiochemical properties such as molecular weight (Mwt), amino acid composition (AA), theoretical isoelectric point (pI), number of negative residues (–R), number of positive residues (+R), extinction coefficient (EC), half-life (h), instability index (II),aliphatic index (AI), and grand average of hydropathy (GRAVY) of the protein sequence (37).

#### Secondary structure prediction

The secondary structure of protein was predicted by using SOPMA and GOR IV. The self-optimized prediction method (SOPMA) describes the three states of the protein structure (helices, turns, and coils). SOPMA predicts 90% of secondary structural information of proteins and it works under the homologous method and predicts 69.5% of amino acids for three states of the secondary structure. SOPMA is mainly classified into four steps. Step one involves the retrieval of homologous protein from UniProt. In step two, alignments of sequence compose the set of homologous proteins. Step three executes the SOPMA method with each and every aligned sequence. In the final step, the conformational state yielding the highest score is attributed to the given amino acid with the averaged conformational score (14).

GOR IV (Garnier-Osguthorpe–Robson) is another method to predict the secondary structure. In version I, GOR has information from the hydrophobic triplet. Hydrophobic triplet information does not significantly improve the predictive power (15). The method GOR IV is formed on information theory; GOR has a mean accuracy of 64.4% for a three state prediction when compared to another version. Version IV is more accurate. The GOR IV method analyzes the secondary structure of the protein and correlates it with net values of each amino acid position and three states (helices, turns, coils) (16).

#### Tertiary structure prediction

The tertiary structure of vlhA genes was constructed using the homology modeling server RaptorX (http://raptorx.uchicago.edu/) and I-TASSER server (https://zhanglab.ccmb.med.umich.edu/I-TASSER/) (17). Raptor X distinguishes itself from other servers by the quality of the alignment between a target sequence and one or multiple distantly related template proteins and by a novel nonlinear scoring function and a probabilistic-consistency algorithm. The predicted tertiary models can be used for binding site and epitope prediction; another application is found to be determining the binding topology of small ligand molecules to putative binding sites on the domain structure generated (54). The I-TASSER server employs *ab initio* modeling to predict 3D structures. The tertiary structures modeled by I-TASSER were subjected to refinement by the GalaxyRefine server (http://galaxy.seoklab.org/cgi-bin/submit.cgi?type=REFINE) (18). This server replaces amino acids with high-probability rotamers and applies molecular dynamic simulation for overall structural relaxation.

#### Structure validation

The refined structure was validated by PROCHECK (https://servicesn.mbi.ucla.edu/PROCHECK/), which analyzes the stereochemical quality of a protein structure by analyzing residue-by–Residue geometry and overall structure geometry (19).

QMEAN is used to analyze the quality of computationally predicted proteins. It is based on two distance-dependent interaction potentials of mean force, C-β atoms and is used to assess long–Range interactions (secondary structure dependent and torsion angle potential dependent). The QMEAN4 score is a linear combination of four statistical potential terms. It is trained to predict the IDDT (The Local Distance Difference Test) score in the range [0, 1]. To calculate the QMEAN Z-score, the normalized raw scores of a given model are compared with scores obtained for a representative set of high resolution X–Ray structures of similar size against the PDB reference set (20–22).

### Functional analysis

#### Subcellular localization prediction

(A) PSLPRED
  PSLpred is used for predicting the subcellular localization of prokaryotic proteins with an overall accuracy of 91.2%. It is a hybrid approach-based method. The prediction accuracies of 90.7, 86.8, 90.3, 95.2, and 90.6% were attained for cytoplasmic, extracellular, inner membrane, outer membrane, and periplasmic proteins, respectively (23).
(B) PSORTB
  PSORTB is the most precise bacterial SCL (subcellular localization) prediction software that was introduced in 2005 and has been widely used. It provides quick and inexpensive means for gaining insight into the protein function, verifying experimental results, annotating newly sequenced bacterial genomes, detecting cell surface/drug targets, and identifying biomarkers for microbes. As a result, only ~50% of proteins encoded in gram-negative bacterial genomes and ~75% of proteins encoded in gram-positive bacterial genomes receive a prediction from PSORTb (24).
(C) CELLO2GO
  CELLO2GO is a publicly available, web-based system for screening various properties of a targeted protein and its subcellular localization. It shows the exact location of the protein. CELLO2GO should be a useful tool for research involving complex subcellular systems because it combines CELLO and BLAST into one form (25).

#### Transmembrane helix prediction

(A) SOSUI
  SOSUI is used for the discrimination of membrane proteins and soluble proteins and the prediction of the transmembrane helix, the accuracy of prediction was 99%, and the corresponding value for the transmembrane helix prediction was 97% (26).
(B) HMMTOP
  A hidden Markov model with special architecture was developed to search transmembrane topology corresponding to the maximum likelihood among all the possible topologies of a given protein. The method is based on the hypothesis that the transmembrane segments and the topology are determined by the difference in the amino acid distributions in various structural parts of these proteins (27).
(C) TMHMM
  TMHMM is a widely used bioinformatics tool, based on the hidden Markov model, which is used to predict transmembrane helices of integral membrane proteins. It is used to predict the number of transmembrane helices and discriminate between soluble and membrane proteins with a high degree of accuracy (28).

#### Signal peptide prediction

(A) Signal p
  Signal p was the first publicly available method to predict signal peptide and its cleavage sites. It is based on deep neural network-based method combined with conditional random field classification and optimized transfer learning for improved signal peptide prediction. The input is given in FASTA format. The server predicts the presence of signal peptides, TAT signal peptides, and lipoprotein signal peptides from proteins present in Archaea, gram-positive bacteria, gram-negative bacteria, and eukaryotes (29).
(B) Target p
  The target p server is used to predict the presence of signal peptides, and mitochondrial transit peptides and others were predicted using the FASTA sequence of the protein (30).

#### Identification of functional domain

The functional domain analysis was carried out using five publicly available tools (CDD-BLAST, HmmScan, Pfam, SCANPROSITE, and SMART). CDD-BLAST annotates the vlhA proteins by generating alignment models of the representative sequence fragment which were in agreement with domain boundaries as observed protein models in NCBI's Conserved Domain Database (31). HmmScan and SMART took a query sequence and searched it against the Pfam profile HMM library as a target database (32–34). Pfam was used to classify vlhA proteins functional families based on similarity (34). To predict the protein function, SCANPROSITE detects homologs and matches against signature from the PROSITE database (35).

## Results

### Phylogenetic analysis

**Figure 2:**
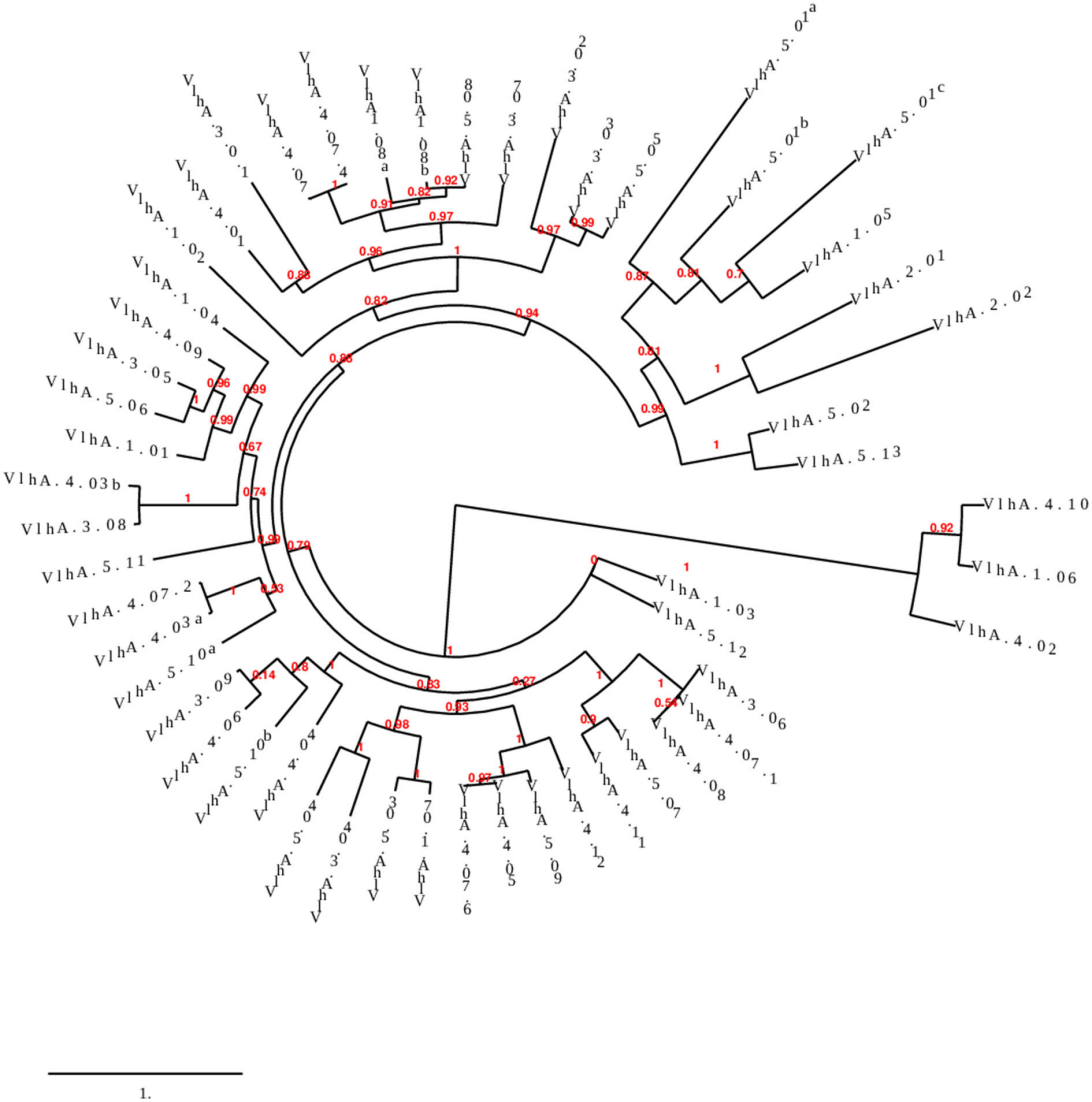
Phylogenetic tree showing the evolutionary relationship of different *M. gallisepticum* vlhA proteins. The numbers indicate bootstrap percentages and the scale indicates the divergence time.

Phylogenetic analysis was performed to examine the differences and relatedness among the vlhA proteins. A phylogenetic tree was constructed by using Phylogeny.fr. The computed data indicated that the expression of vlhA proteins during the course of infection varies greatly and vlhA from the five loci here clustered into different groups. The bootstrap values in the phylogenetic tree created for *M. gallisepticum* vlhA proteins showed that the proteins had less evolutionary similarity (Figure 2), and the divergence in sequence during evolution may have developed to evade host immune response and to adapt to each host. As a consequence, each protein has evolved due to strain during the course of infection, thus leading to antigenic variation (36).

### Structural analysis

#### Physiochemical characterization

**Figure 3:**
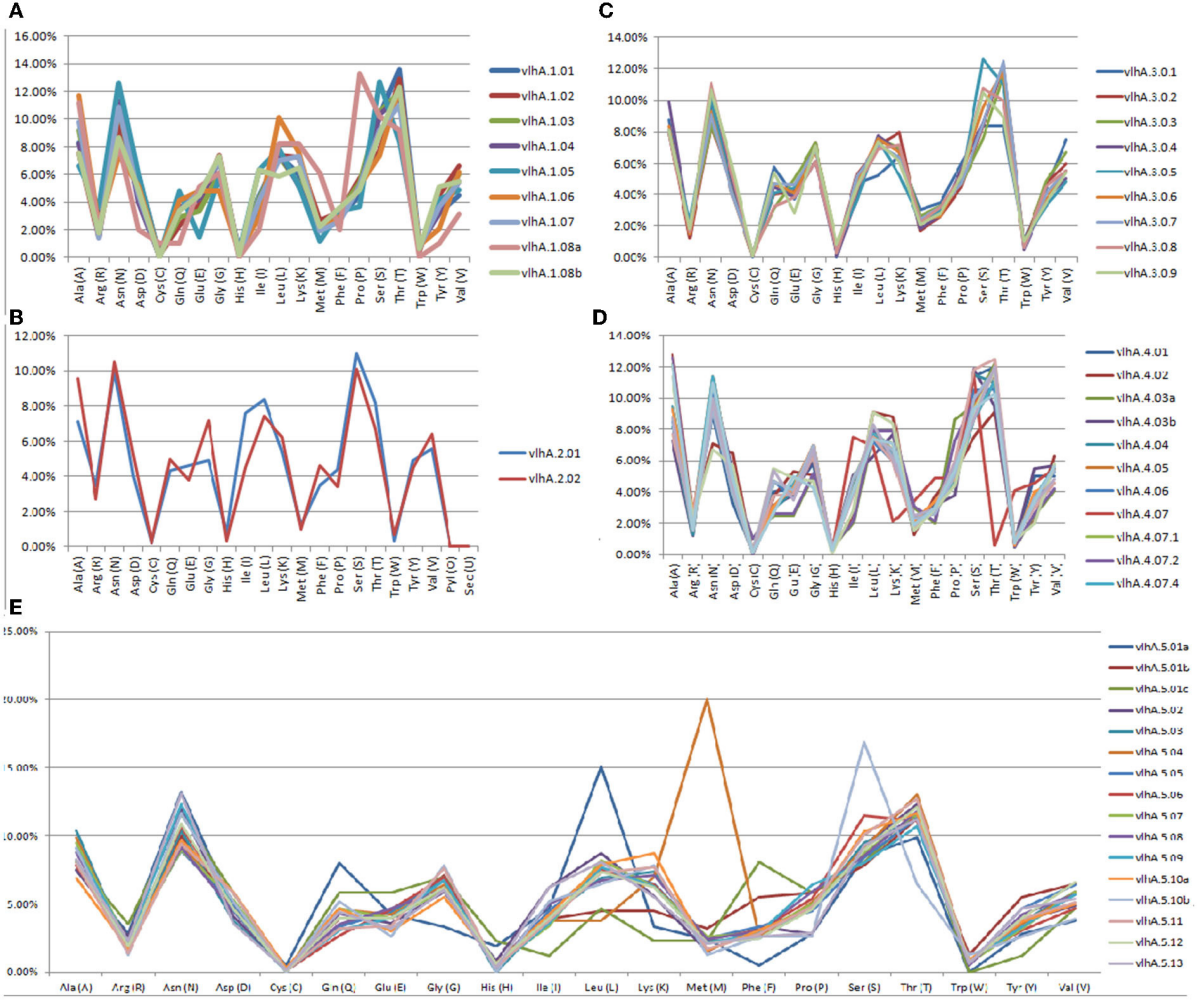
Graphical representation of amino acid composition of *M. gallisepticum* vlhA proteins. **(A)** vlhA 1 group, **(B)** vlhA 2 group, **(C)** vlhA 3 group, **(D)** vlhA 4 group, and **(E)** vlhA group 5.

The ExPASy ProtParam was employed to analyze the protein primary structures and compute different parameters for their physiochemical properties. The number of amino acid residues in vlhA proteins varied from 77 to 795 amino acids. The composition of amino acid residues in each vlhA protein is presented in Figure 3. The molecular weight of these proteins varied from 8.12 to 85.3 kDa. The pI values of these proteins range from acidic pI 4.63 to alkaline pI 9.21. If the instability index (II) is above 40, the protein was considered to be unstable. As shown in Table 3, except a few vlhA (vlhA.1.08, vlhA.2.01, vlhA.5.01c, and vlhA.5.10b) proteins, other proteins were considerably stable. The aliphatic index (AI) of these vlhA proteins varied from 27.86 to 95.75. The high AI values indicated the thermal stability and hydrophobic nature of the proteins. When a protein was found to have a greater negative grand average of hydropathy (GRAVY) values, it indicated the hydrophilic nature of the protein and the possibility of better interactions between the protein and water (37). The complete physicochemical analysis of all the vlhA proteins is listed in Table 3.

**Table 3:** Physiochemical properties like number of amino acids, molecular weight, isoelectric point, extinction coeffcient, half-life (h), instability index, aliphatic index, and GRAVY of *M. gallisepticum* vlhA proteins.

**Protein name**	**Amino acid**	**Mol.wt**	**PI**	**Extinction coefficient**	**half Life (h)**	**instability Index**	**Aliphatic index**	**GRAVY**
vlhA.1.01	686	74.02	6.23	71,280	30	26.86	67.46	−0.542
vlhA.1.02	666	71.65	5.3	61,200	30	25.89	68.83	−0.445
vlhA.1.03	682	72.83	5.54	63,260	30	28.14	71.85	−0.385
vlhA.1.04	697	74.83	6.81	65,780	30	32.57	68.75	−0.5
vlhA.1.05	730	79.70	9.08	73,690	30	36.44	36.44	−0.525
vlhA.1.06	754	80.92	6.36	62,340	30	27.55	80.44	−0.329
vlhA.1.07	728	77.55	5.49	67,730	30	30.03	69.08	−0.513
vlhA.1.08	98	10.21	9.25	1,490	30	46.91	59.9	−0.446
vlhA.1.08b	494	53.55	5.28	53,750	30	24.5	70.71	−0.414
vlhA.2.01	607	66.88	8.19	55,700	30	41.99	85.65	−0.354
vlhA.2.02	582	63.10	6.79	60,740	30	29.80	74.30	−0.430
vlhA.3.0.1	536	58.00	5.28	67,270	30	26.83	68.97	−0.442
vlhA.3.02	646	69.75	8.37	77,700	30	24.51	73.85	−0.439
vlhA.3.03	645	69.93	5.38	68,190	30	27.6	73.35	−0.389
vlhA.3.04	734	78.52	5.68	62,230	30	26.34	69.73	−0.515
vlhA.3.05	708	75.77	5.36	72,770	30	37.21	65.9	−0.531
vlhA.3.06	688	73.76	6.8	72,250	30	30.51	72.63	−0.427
vlhA.3.07	656	70.87	5.78	60,740	30	231.64	72.15	−0.426
vlhA.3.08	692	74.76	6	68,190	30	33.47	68.4	−0.537
vlhA.3.09	707	76.06	5.68	74,260	30	30.91	69.99	−0.55
vlhA.4.01	644	69.49	8.74	69,680	30	24.79	70.51	−0.415
vlhA.4.02	751	80.74	5.76	62,340	30	27.23	76.11	−0.438
vlhA.4.03a	197	20.71	9.06	13,075	30	24.19	61.57	−0.525
vlhA.4.03b	506	55.11	6.25	63,720	30	33.38	68.64	−0.523
vlhA.4.04	679	72.64	5.60	70,250	30	26.79	71.72	−0.465
vlhA.4.05	673	72.27	6.01	67,270	30	25.63	71.66	−0.466
vlhA.4.06	698	74.98	5.56	74,260	30	30.59	66.85	−0.545
vlhA.4.07	667	71.81	8.72	62,230	30	30.34	67.80	−0.501
vlhA.4.07.1	684	73.2	7.56	70,250	30	30.66	72.35	−0.424
vlhA.4.07.2	191	20.1	9.21	13,075	30	24.54	63.51	−0.493
vlhA.4.07.4	673	72.3	6.32	68,760	30	25.37	71.66	−0.463
vlhA.4.07.6	667	71.7	8.30	62,230	30	29.85	67.80	−0.496
vlhA.4.08	688	73.6	7.56	70,250	30	30.54	71.93	−0.428
vlhA.4.09	710	76	6.88	69,790	30	31.84	65.17	−0.514
vlhA.4.10	795	85.3	7.52	62,340	30	30.29	74.34	−0.479
vlhA4.11	690	74	6.40	61,770	30	28.52	65.82	−0.544
vlhA.4.12	701	75.1	5.28	63,260	30	27.86	27.86	−0.446
vlhA.5.01a	212	23.32	5.10	8,940	30	38.68	95.75	−0.456
vlhA.5.01b	309	33.93	4.80	47,330	30	31.19	59.35	−0.431
vlhA.5.01c	86	9.38	4.63	1,490	30	41.87	44.30	−0.779
vlhA.5.02	610	66.45	8.51	56,270	30	30.65	79.98	−0.407
vlhA.5.03	728	77.47	8.78	67,730	30	28.91	72.15	−0.449
vlhA.5.04	740	78.90	5.17	66,240	30	35.27	69.27	−0.467
vlhA.5.05	644	69.83	5.73	66,700	30	26.68	73.93	−0.392
vlhA.5.06	703	75.28	5.75	65,780	30	28.52	65.82	−0.544
vlhA.5.07	681	73.27	5.55	60,280	30	30.09	71.82	−0.444
vlhA.5.08	661	71.41	6.32	58,220	30	29.53	70.88	−0.460
vlhA.5.09	701	75.19	6.42	67,270	30	24.61	69.83	−0.531
vlhA.5.10a	642	70.06	9.04	68,885	30	26.23	68.69	−0.619
vlhA.5.10b	77	8.12	8.03	8,480	30	51.99	65.97	−0.619
vlhA.5.11	711	75.88	6.87	69,220	30	20.48	66.03	−0.55
vlhA.5.12	678	73.12	5.81	67,730	30	25.23	71.80	−0.483
vlhA.5.13	616	66.94	8.89	65,210	30	29.03	79.53	−0.394

#### Secondary structure prediction

The secondary structure of vlhA proteins was predicted using SOPMA and GOR IV servers that showed similar results where the percentage of random coils was higher when compared with alpha helices and extended turns (Supplementary Table 2). Previous studies reported that the presence of a higher percentage of random coil structures in bacterial proteins facilitated the dimerization and/or colocalization process and also act as adaptor proteins (38–43).

#### Three-dimensional structure modeling and validation

**Figure 4:**
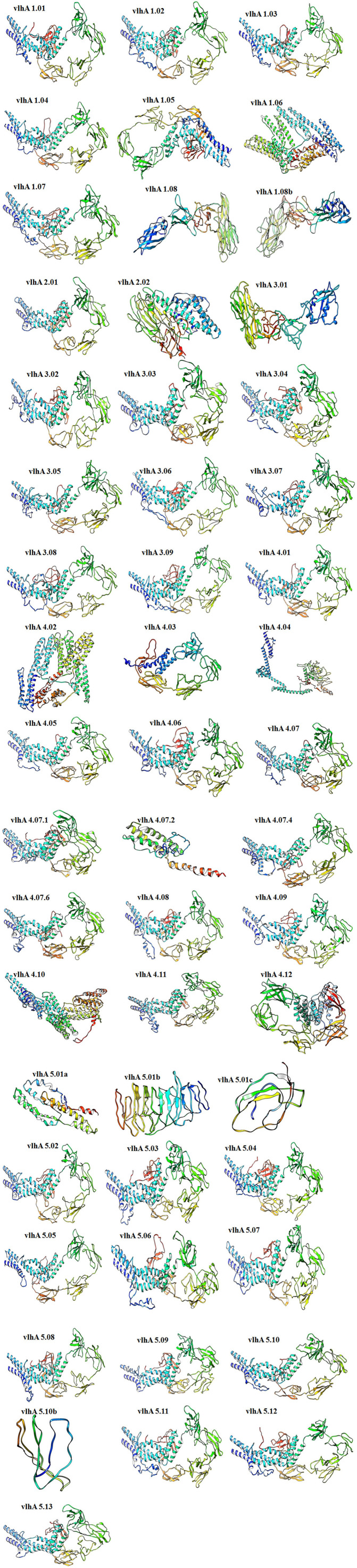
Three-dimensional *ab initio* models of vlhA proteins. Visualizations of model structures were performed by UCSF Chimera.

The tertiary models of vlhA proteins were constructed using the server called RaptorX and I Tasser. In tertiary models predicted by Raptor X, the number of amino acids was less compared to the input sequence, and thus the model predicted by I-TASSER was used for further analysis. The results from I-Tasser are consistent with the secondary structure prediction where these proteins were predicted to have a high percentage of random coil structures (Figure 4).

The PDBsum-PROCHECK program was used to validate the constructed three-dimensional models of these proteins. The Ramachandran Plot was used in the PROCHECK program to present the backbone conformation of proteins. The predicted models of vlhA proteins were analyzed and majority of the amino acid residues fall in the favored and allowed regions of the Ramachandran plot which indicates the good quality of the predicted models (Table 4).

**Table 4:** Tertiary structural validation- Qmean Score, Ramachandran plot most favored region and functional analysis-Subcellular Localization, Transmembrane helix, Signal peptide of vlhA proteins.

**S.No**	**Protein name**	**Qmean score**	**Ramachandran plot most favored region**	**Subcellular localization**	**Transmenbrane helix**	**Signal peptide**
1	vlhA.1.01	−10.78	68.1%	Extracellular	2(44–61)(106–123)	Yes
2	vlhA.1.02	−10.06	70.2%	Extracellular	2(42–59)(104–121)	Yes
3	vlhA.1.03	−9.32	69.3%	Extracellular	2(42–59)(104–121)	Yes
4	vlhA.1.04	−10.62	69.6%	Extracellular	2(42–59)(104–121)	Yes
5	vlhA.1.05	−11.63	66.3%	Extracellular	0	Yes
6	vlhA.1.06	−7.45	82.0%	Periplasmic	2(42–59)(104–121)	Yes
7	vlhA.1.07	−10.65	68.4%	Extracellular	2(42–59)(104–121)	No
8	vlhA.1.08	−12.84	65.5%	Periplasmic	2(66–83)(126–146)	Yes
9	vlhA.1.08b	−12.84	65.5%	Extracellular	0	Yes
10	vlhA.2.01	−10.16	66.4%	Extracellular	0	Yes
11	vlhA.2.02	−9.48	69.0%	Extracellular	2(42–59)(104–121)	No
12	vlhA.3.0.1	−14.31	52.8%	Periplasmic	0	Yes
13	vlhA.3.02	−9.78	67.1%	Extracellular	0	Yes
14	vlhA.3.03	−10.31	68.0%	Extracellular	2(46–63)(108–125)	No
15	vlhA.3.04	−10.86	66.4%	Extracellular	2(46–63)(108–125)	Yes
16	vlhA.3.05	−10.04	69.3%	Extracellular	2(46–63)(108–125)	Yes
17	vlhA.3.06	−11.47	65.8%	Extracellular	1(9–26)	Yes
18	vlhA.3.07	−11.57	62.5%	Extracellular	1(9–26)	Yes
19	vlhA.3.08	−10.78	66.5%	Extracellular	1(9–26)	Yes
20	vlhA.3.09	−9.09	66.2%	Extracellular	1(9–26)	Yes
21	vlhA.4.01	−9.02	69.1%	Extracellular	1(9–26)	No
22	vlhA.4.02	−7.77	81.5%	Periplasmic	1(9–26)	Yes
23	vlhA.4.03a	−10.23	66.1%	Outermenbrane	1(9–26)	Yes
24	vlhA.4.03b	−12.26	66.3%	Extracellular	0	Yes
25	vlhA.4.04	−12.23	60.7%	Extracellular	2(44–61)(106–123)	Yes
26	vlhA.4.05	−10.63	66.3%	Extracellular	2(44–61)(106–123)	Yes
27	vlhA.4.06	−10.28	67.5%	Extracellular	2(44–61)(106–123)	Yes
28	vlhA.4.07	−10.81	66.7%	Extracellular	2(44–61)(106–123)	Yes
29	vlhA.4.07.1	−11.19	67.6%	Extracellular	2(46–63) (108–125)	Yes
30	vlhA.4.07.2	−11.85	60.6%	Extracellular	2(66–83) (129–146)	Yes
31	vlhA.4.07.4	−9.15	68.2%	Extracellular	2(46–63) (108–125)	Yes
32	vlhA.4.07.6	−10.71	67.4%	Extracellular	2(46–63) (108–125)	Yes
33	vlhA.4.08	−10.65	65.5%	Extracellular	3(10–27) (44–61) (106–123)	Yes
34	vlhA.4.09	−11.45	66.5%	Outermenbrane	2(44–61) (106–123)	Yes
35	vlhA.4.10	−7.86	81.4%	Periplasmic	2(44–61) (106–123)	Yes
36	vlhA4.11	−10.76	65.6%	Extracellular	2(44–61) (106–123)	Yes
37	vlhA.4.12	−10.43	67.9%	Outermenbrane	2(44–61) (106–123)	Yes
38	vlhA.5.01a	−12.07	56.3%	Extracellular	0	Yes
39	vlhA.5.01b	−13.51	40.4%	Extracellular	0	Yes
40	vlhA.5.01c	−8.69	38.4%	Extracellular	0	Yes
41	vlhA.5.02	−10.15	67.0%	Extracellular	0	Yes
42	vlhA.5.03	−11.14	69.7%	Extracellular	2(44–61)	Yes
43	vlhA.5.04	−10.63	70.0%	Extracellular	2(44–61)(106–123)	Yes
44	vlhA.5.05	−9.17	69.0%	Extracellular	2(44–61)(106–123)	Yes
45	vlhA.5.06	−9.65	67.1%	Extracellular	1 (19–38)	Yes
46	vlhA.5.07	−10.58	67.1%	Extracellular	2(44–61) (106–123)	Yes
47	vlhA.5.08	−12.05	66.6%	Extracellular	2(44–61) (106–123)	Yes
48	vlhA.5.09	−11.32	66.4%	Extracellular	2(44–61) (106–123)	Yes
49	vlhA.5.10a	−9.33	70.1%	Extracellular	2(64–81)(127–144)	Yes
50	vlhA.5.10b	−11.02	25.4%	Outermenbrane	0	Yes
51	vlhA.5.11	−11.26	67.6%	Extracellular	2(44–61)(106–123)	Yes
52	vlhA.5.12	−11.73	71.3%	Extracellular	2(44–61)(106–123)	Yes
53	vlhA.5.13	−10.82	66.4%	Extracellular	0	Yes

QMEAN z-score was used to validate the good quality of these predicted tertiary models. This QMEAN software determined the closeness and similarity of the computationally predicted model with the existing PDB reference set. The normalized QMEAN score is provided in Table 4.

### Functional analysis

#### Localization of vlhA proteins

In this study, 3 different servers (CELLO2GO, PSORTB, and PSLPRED) were used to predict the cellular location of vlhA proteins. As provided in Table 4, the vlhA proteins were predicted to be extracellular proteins which help in the host interactions and immune evasion. The results were similar for all the three servers. TMMHMM, HMMTOP, and SOSUI servers were used to predict the presence of transmembrane helices in these proteins. Except vlhA-−1.08b, 2.01, 2.02, 3.01, 3.02, 3.08, 4.01, 4.03b, 5.01a, 5.01b, 5.01c, 5.02, 5.08, 5.10b, and 5.13, other proteins were predicted to have transmembrane helices (Table 4). The prediction results are consistent among the servers. Based on the prediction using SignalP and TargetP servers, several vlhA proteins having lower values indicated the absence of signal peptides in them. In contrast, the vlhA proteins with higher values indicated the presence of signal peptides in their sequence (Table 4).

#### Identification of the functional domain

There are a large number of proteins that have no assigned function. For those proteins, the annotation generally depends on the sequence homology techniques (21). Functional domains were identified using CDD- BLAST, HmmScan, Pfam, SCANPROSITE, and SMART publicly available tools. After screening the vlhA proteins in the above mentioned servers, all the proteins were grouped under the mycoplasma hemagglutinin family by all the servers. Based on the similarity of the sequences of these proteins with mycoplasma hemagglutinin, these proteins were predicted to play a role in the hemagglutination process. The mycoplasma hemagglutinin family consists of several hemagglutinin sequences from mycoplasma species. The major plasma membrane proteins, vlhAs, of *M. gallisepticum* are cell adhesions or hemagglutinin molecules. The hemagglutination process of mycoplasma plays a crucial role in host immune evasion; the exact mechanism through which the hemagglutination mediated immune evasion occurs is yet to be explored (44, 45).

## Discussion

Variable lipoprotein hemagglutinin A gene encodes immunodominant proteins that are believed to be responsible for *M. gallisepticum'*s host cell interaction, pathogenesis, and immune evasion; however, their exact mechanism is unknown (46). The sound knowledge about the mechanism of immune evasion by this protein family will be valuable in the development of drugs and vaccines against *M. gallisepticum* infection in chickens. Protein structure and function identification is an essential step for understanding its cellular and molecular processes. *In silico* homology modeling studies provide an opportunity to establish a route for the structural modeling and analysis of vlhA proteins. With rapid advances in bioinformatics and computational biology, the prediction and validation of the structure and function of proteins have become easily accessible. The importance of functional analysis of proteins includes deeper knowledge in molecular mechanisms of disease progression, exploration of effective prophylactic targets, relationship, and interaction with other proteins in the same microorganism.

This study has analyzed the vlhA proteins from *M. gallisepticum* strain R low for its structural and functional characteristics. The amino acid sequences of vlhA proteins were retrieved in FASTA format from the UniProt database and used for further structural and functional analyses. The physiochemical characteristics such as amino acid composition, isoelectric point (pI), number of negative and positive residues, extinction coefficient, half-life, instability index (II), aliphatic index (AI), and grand average of hydropathy (GRAVY) of these proteins were predicted. According to the results obtained, a higher number of amino acids such as threonine, asparagine, serine, and alanine were observed whereas the amino acids such as cysteine, histidine, and tryptophan were low in amount. Cysteines are important for the formation of disulfide bonds in the protein structure which cannot be easily substituted or replaced and often acts together with histidines which are commonly present in the active or binding sites of the proteins (38). These vlhA proteins have the average molecular weight of 59.28 kDa, and are hydrophilic in nature and stable. The secondary structure of these proteins contains a higher percentage of random coils which are believed to facilitate in the dimerization and/or colocalization process and may also act as adaptor proteins (39–43, 53). The tertiary structures of vlhA proteins were predicted and validated for the good quality of the computationally predicted protein structure. These proteins have been predicted to be stable with the higher percentage of amino acids present in the most favored regions (>80%). The obtained QMEAN score indicated the good quality of these proteins with higher QMEAN values (20). As for the functional prediction of vlhA proteins, all of these proteins were predicted to be extracellular which may subsequently help in the immune evasion of the *M. gallisepticum* from the host immune system. The identification of the functional domain was performed by the sequence homology techniques. The result obtained showed that the domains of these proteins were similar to the mycoplasma hemagglutinin family as they consist of hemagglutinin sequences from the mycoplasma family and predicted to be involved in the hemagglutination process. It has been reported that the genetic determinants that code for the hemagglutinins are organized into a large family of genes and that only one of these genes is predominately expressed during the course of infection at a given time (44, 47–49). Antigenic variation or phenotypic switching occurs due to high frequency genetic mutations. Due to the lack of a rigid cell wall, the lipoproteins in the mycoplasma cell membrane function as the major elements that come into contact with the host environment (45, 46, 50). These proteins undergo antigenic variation through on/off switching, domain shuffling, and size variation to modify the antigenic components on their cell surface to produce heterotypes that allow mycoplasma to evade recognition and clearance by host immune cells that largely eliminate homo-types. Numerous human and animal mycoplasma species have the ability to go through antigenic variation so that these bacteria can evade recognition by the host humoral immune system (51, 52). In *M. gallisepticum*, the hemagglutination process may play a role in triggering the antigenic variation cascade leading to immune evasion. Since the exact function and machinery of these vlhA proteins are not determined at present, the *in silico* structural and functional prediction of these proteins may help in the determination of its cellular and molecular processes. To the best of our knowledge, this is the first study to explore the structural and functional properties of vlhA proteins. These findings may aid in understanding the mechanism of immune evasion by vlhA proteins.

## Conclusion

Identifying the molecular processes by which the vlhA protein evades the host immune response is critical in understanding the pathogenicity of *M. gallisepticum* and will aid in the development of efficient infection control measures. *In silico* homology modeling studies allow researchers to build a pipeline for structural modeling and functional analysis of any protein as part of discovering the molecular mechanism of the protein's function and therapeutic targets. The physicochemical features of selected vlhA that are important for immune evasion were given in this work. The study also included secondary structure and tertiary model characteristics for the vlhA proteins. Furthermore, the functional analysis revealed that the vlhA proteins are clustered under the mycoplasma hemagglutinin family. For functional analysis of vlhA proteins, multiple servers like CDD- BLAST, HmmScan, Pfam, SCANPROSITE, and SMART were used and all the servers grouped the vlhA proteins under the mycoplasma hemagglutinin family; the results obtained were consistent, thus validating the uniqueness of our findings. The significance of this study is the analysis and exploration of unknown structural and functional characteristics of vlhA proteins through the application of latest bioinformatics software like Protparam, I Tasser, PSORTB, TMMHMM, SignalP, and Pfam, thus bridging the gap in knowledge in the role of vlhA proteins in *M. gallisepticum* pathogenesis. This research will serve as a foundation for future experimental studies aimed at clarifying the functional molecular mechanism of immune response.

## Data availability statement

The original contributions presented in the study are included in the article/Supplementary material, further inquiries can be directed to the corresponding author/s.

## Author contributions

SM and MH designed and performed the experimental studies. SM carried out the *in silico* experiments. The manuscript was written by SM and MH. Both authors contributed to the article and approved the submitted version.

## Conflict of interest

The authors declare that the research was conducted in the absence of any commercial or financial relationships that could be construed as a potential conflict of interest.

## Publisher's note

All claims expressed in this article are solely those of the authors and do not necessarily represent those of their affiliated organizations, or those of the publisher, the editors and the reviewers. Any product that may be evaluated in this article, or claim that may be made by its manufacturer, is not guaranteed or endorsed by the publisher.
