# Supplementary material for: In silico structural homology modeling and functional characterization of Mycoplasma gallisepticum variable lipoprotein hemagglutin proteins
Source: Front Vet Sci. 2022 Aug 4;9:943831. doi: 10.3389/fvets.2022.943831 (PMC9386052; doi:10.3389/fvets.2022.943831)
Supplement: Supplementary file 1 [file Table_1.DOCX]

| **S.No** | **Protein name** | **Accession No.** |
| --- | --- | --- |
|  | vlhA.1.01 | D3DEJ8 |
|  | vlhA.1.02 | Q7NB57 |
|  | vlhA.1.03 | Q7NB56 |
|  | vlhA.1.04 | Q7NB55 |
|  | vlhA.1.05 | Q7NB54 |
|  | vlhA.1.06 | Q7NB52 |
|  | vlhA.1.07 | Q7NB51 |
|  | vlhA.1.08a | D3DEK1 |
|  | vlhA.1.08b | D3DEK0 |
|  | vlhA.2.01 | Q7NB25 |
|  | vlhA.2.02 | Q7NB24 |
|  | vlhA.3.0.1 | D3DELO |
|  | vlhA.3.02 | Q7NAP5 |
|  | vlhA.3.03 | Q7NAP4 |
|  | vlhA.3.04 | Q7NAP3 |
|  | vlhA.3.05 | Q7NAP2 |
|  | vlhA.3.06 | Q7NAP1 |
|  | vlhA.3.07 | Q7NAP0 |
|  | vlhA.3.08 | Q7NAN9 |
|  | vlhA.3.09 | Q7NAN8 |
|  | vlhA.4.01 | Q7NBR8 |
|  | vlhA.4.02 | Q7NBR7 |
|  | vlhA.4.03a | D3DEH6 |
|  | vlhA.4.03b | D3DEH7 |
|  | vlhA.4.04 | Q7NBR6 |
|  | vlhA.4.05 | Q7NBR5 |
|  | vlhA.4.06 | Q7NBR4 |
|  | vlhA.4.07 | Q7NBR3 |
|  | vlhA.4.07.1 | D3DEH8 |
|  | vlhA.4.07.2 | D3DEH9 |
|  | vlhA.4.07.4 | D3DEI2 |
|  | vlhA.4.07.6 | D3DEI4 |
|  | vlhA.4.08 | Q7NBR2 |
|  | vlhA.4.09 | Q7NBR1 |
|  | vlhA.4.10 | Q7NBR0 |
|  | vlhA.4.11 | Q7NBQ9 |
|  | vlhA.4.12 | Q7NBQ8 |
|  | vlhA.5.01a | Q7NBE3 |
|  | vlhA.5.01b | D3DEJ2 |
|  | vlhA.5.01c | D3DEJ3 |
|  | vlhA.5.02 | Q7NBE2 |
|  | vlhA.5.03 | Q7NBE1 |
|  | vlhA.5.04 | Q7NBE0 |
|  | vlhA.5.05 | Q7NBD9 |
|  | vlhA.5.06 | Q7NBD8 |
|  | vlhA.5.07 | Q7NBD7 |
|  | vlhA.5.08 | Q7NBD6 |
|  | vlhA.5.09 | Q7NBD5 |
|  | vlhA.5.10a | Q7NBD4 |
|  | vlhA.5.10b | D3DEJ4 |
|  | vlhA.5.11 | Q7NBD3 |
|  | vlhA.5.12 | Q7NBD2 |
|  | vlhA.5.13 | Q7NBD0 |

**Supplementary** **Table 1:** vlhA proteins and their UniProt Ids

| **PROTEIN NAME** | **ALPHA HELIX** | **RANDOM COIL** | **EXTENDED STRAND** | **SERVERS** |
| --- | --- | --- | --- | --- |
| vlhA.1.01 | 27.11% | 53.35% | 19.53% | SOPMA |
|  | 22.89% | 53.35% | 23.76% | GOR IV |
| vlhA.1.02 | 29.28% | 46.85% | 21.02% | SOPMA |
|  | 16.37% | 60.96% | 22.67% | GOR IV |
| vlhA.1.03 | 26.98% | 52.20% | 20.82% | SOPMA |
|  | 20.38% | 57.33% | 22.29% | GOR IV |
| vlhA.1.04 | 24.68% | 54.09% | 21.23% | SOPMA |
|  | 18.36% | 60.69% | 20.95% | GOR IV |
| vlhA.1.05 | 26.44% | 48.90% | 22.05% | SOPMA |
|  | 18.49% | 57.81% | 23.70% | GOR IV |
| vlhA.1.06 | 39.92% | 38.20% | 17.64% | SOPMA |
|  | 39.79% | 44.96% | 15.25% | GOR IV |
| vlhA.1.07 | 26.51% | 49.45% | 21.15% | SOPMA |
|  | 26.37% | 52.34% | 20.33% | GOR IV |
| vlhA.1.08 a | 33.67% | 46.94% | 14.29% | SOPMA |
|  | 22.45% | 58.16% | 19.39% | GOR IV |
| vlhA.1.08 b | 19.43% | 50.61% | 26.32% | SOPMA |
|  | 13.97% | 55.06% | 30.97% | GOR IV |
| vlhA.2.01 | 30.15% | 45.80% | 24.05% | SOPMA |
|  | 27.02% | 52.72% | 20.26% | GOR IV |
| vlhA.2.02 | 29.38% | 47.08% | 23.54% | SOPMA |
|  | 34.54% | 46.39% | 19.07% | GOR IV |
| vlhA.3.0.1 | 16.23% | 56.53% | 23.88% | SOPMA |
|  | 16.79% | 54.85% | 28.36% | GORIV |
| vlhA.3.02 | 30.34% | 45.04% | 21.36% | SOPMA |
|  | 25.85% | 50.00% | 24.41% | GOR IV |
| vlhA.3.03 | 27.13% | 50.85% | 22.02% | SOPMA |
|  | 27.29% | 48.84% | 23.88% | GOR IV |
| vlhA.3.04 | 25.89% | 55.04% | 19.07% | SOPMA |
|  | 31.47% | 49.86% | 18.66% | GOR IV |
| vlhA.3.05 | 27.54% | 49.29% | 20.20% | SOPMA |
|  | 20.34% | 56.36% | 23.31% | GOR IV |
| vlhA.3.06 | 28.05% | 52.62% | 19.33% | SOPMA |
|  | 30.09% | 47.24% | 22.67% | GOR IV |
| vlhA.3.07 | 27.74% | 51.07% | 21.19% | SOPMA |
|  | 25.46% | 49.85% | 24.70% | GOR IV |
| vlhA.3.08 | 26.01% | 54.48% | 19.51% | SOPMA |
|  | 21.53% | 54.91% | 23.55% | GOR IV |
| vlhA.3.09 | 24.61% | 55.16% | 20.23% | SOPMA |
|  | 25.04% | 54.46% | 20.51% | GOR IV |
| vlhA.4.01 | 28.46% | 49.92% | 21.62% | SOPMA |
|  | 22.24% | 54.74% | 23.02% | GOR IV |
| vlhA.4.02 | 43.01% | 37.82% | 14.25% | SOPMA |
|  | 53.81% | 44.07% | 12.78% | GOR IV |
| vlhA.4.03a | 53.81% | 39.09% | 7.11% | SOPMA |
|  | 37.06% | 50.76% | 12.18% | GOR IV |
| vlhA.4.03b | 19.17% | 53.36% | 25.10% | SOPMA |
|  | 13.44% | 56.92% | 29.64% | GOR IV |
| vlhA 4.04 | 27.39% | 48.75% | 21.35% | SOPMA |
|  | 23.56% | 50.96% | 25.48% | GOR IV |
| vlhA.4.05 | 29.27% | 45.91% | 22.44% | SOPMA |
|  | 27.34% | 47.85% | 24.81% | GOR IV |
| vlhA.4.06 | 28.08% | 47.71% | 20.63% | SOPMA |
|  | 23.50% | 53.58% | 22.92% | GOR IV |
| vlhA.4.07 | 27.14% | 52.47% | 20.39% | SOPMA |
|  | 28.34% | 49.33% | 22.34% | GOR IV |
| vlhA.4.07.1 | 28.07% | 52.63% | 19.30% | SOPMA |
|  | 31.14% | 47.37% | 21.49% | GOR IV |
| vlhA.4.07.2 | 19.63% | 37.17% | 6.81% | SOPMA |
|  | 23.83% | 49.21% | 12.57% | GOR IV |
| vlhA.4.07.4 | 28.08% | 45.91% | 22.59% | SOPMA |
|  | 27.34% | 47.70% | 24.96% | GOR IV |
| vlhA.4.07.6 | 28.49% | 47.38% | 20.69% | SOPMA |
|  | 28.34% | 49.33% | 22.34% | GOR IV |
| vlhA.4.08 | 30.38% | 46.80% | 20.20% | SOPMA |
|  | 30.96% | 47.67% | 21.37% | GOR IV |
| vlhA.4.09 | 25.63% | 54.65% | 19.72% | SOPMA |
|  | 18.03% | 59.30% | 22.68% | GOR IV |
| vlhA.4.10 | 39.37% | 46.29% | 14.34% | SOPMA |
|  | 45.16% | 42.89% | 11.95% | GOR IV |
| vlhA.4.11 | 28.41% | 47.68% | 21.30% | SOPMA |
|  | 25.94% | 53.33% | 20.72% | GOR IV |
| vlhA.4.12 | 25.82% | 53.78% | 20.40% | SOPMA |
|  | 36.38% | 46.65% | 16.98% | GOR IV |
| vlhA.5.01a | 70.28% | 26.42% | 3.30% | SOPMA |
|  | 41.98% | 47.17% | 10.85% | GOR IV |
| vlhA.5.01b | 8.09% | 60.84% | 28.84% | SOPMA |
|  | 10.36% | 59.55% | 30.10% | GOR IV |
| vlhA.5.01c | 11.63% | 62.79% | 23.26% | SOPMA |
|  | 5.81% | 72.09% | 22.09% | GOR IV |
| vlhA.5.02 | 30.00% | 43.44% | 23.44% | SOPMA |
|  | 27.05% | 47.54% | 25.41% | GOR IV |
| vlhA.5.03 | 26.79% | 53.30% | 19.92% | SOPMA |
|  | 27.88% | 50.82% | 21.29% | GOR IV |
| vlhA.5.04 | 24.86% | 56.49% | 18.65% | SOPMA |
|  | 30.27% | 50.68% | 19.05% | GOR IV |
| vlhA.5.05 | 28.73% | 49.53% | 21.74% | SOPMA |
|  | 25.93% | 52.02% | 22.05% | GOR IV |
| vlhA.5.06 | 26.88% | 49.36% | 20.77% | SOPMA |
|  | 20.48% | 57.47% | 22.05% | GOR IV |
| vlhA.5.07 | 26.43% | 52.28% | 21.29% | SOPMA |
|  | 28.34% | 50.51% | 21.15% | GOR IV |
| vlhA.5.08 | 27.84% | 48.11% | 21.63% | SOPMA |
|  | 22.24% | 52.80% | 24.96% | GOR IV |
| vlhA.5.09 | 27.25% | 49.22% | 21.11% | SOPMA |
|  | 27.96% | 50.93% | 21.11% | GOR IV |
| vlhA.5.10a | 29.75% | 50.62% | 19.63% | SOPMA |
|  | 21.96% | 54.21% | 23.83% | GOR IV |
| vlhA.5.10b | 3.90% | 61.04% | 29.87% | SOPMA |
|  | 23.38% | 57.14% | 19.48% | GOR IV |
| vlhA.5.11 | 26.16% | 53.59% | 20.25% | SOPMA |
|  | 19.97% | 56.54% | 23.49% | GOR IV |
| vlhA.5.12 | 26.99% | 52.95% | 20.06% | SOPMA |
|  | 22.71% | 54.13% | 23.16% | GOR IV |
| vlhA.5.13 | 27.44% | 48.70% | 23.86% | SOPMA |
|  | 25.49% | 45.62% | 28.90% | GOR IV |

**Supplementary Table 2:** Prediction of percentage of secondary components using SOPMA & GOR IV servers
